# Non-Mendelian assortment of homologous autosomes of different sizes in males is the ancestral state in the *Caenorhabditis* lineage

**DOI:** 10.1038/s41598-017-13215-4

**Published:** 2017-10-09

**Authors:** Tho Son Le, Fang-Jung Yang, Yun-Hua Lo, Tiffany C. Chang, Jung-Chen Hsu, Chia-Yi Kao, John Wang

**Affiliations:** 1 Biodiversity Research Center, Academia Sinica, Taipei, 11529 Taiwan; 2 Department of Molecular Genetics and Gene Technology, College of Forestry Biotechnology, Vietnam National University of Forestry, Hanoi, Vietnam

## Abstract

Organismal genome sizes vary by six orders of magnitude and appear positively correlated with organismal size and complexity. Neutral models have been proposed to explain the broad patterns of genome size variation based on organism population sizes. In the *Caenorhabditis* genus, hermaphrodite genomes are smaller than those of gonochoristic species. One possible driving force for this genome size difference could be non-random chromosome segregation. In *Caenorhabditis elegans*, chromosome assortment is non-independent and violates Mendel’s second law. In males, the shorter homologue of a heterozygous autosome pair preferentially co-segregates with the X chromosome while the longer one preferentially co-segregates with the nullo-X (O) chromosome in a process we call “skew”. Since hermaphrodites preferentially receive the shorter chromosomes and can start populations independently, their genome size would be predicted to decrease over evolutionary time. If skew is an important driver for genome size reduction in hermaphroditic *Caenorhabditis* species, then it should be present in all congeneric species. In this study, we tested this hypothesis and found that skew is present in all eight examined species. Our results suggest that skew is likely the ancestral state in this genus. More speculatively, skew may drive genome size patterns in hermaphroditic species in other nematodes.

## Introduction

Why is there variation in genome size amongst organisms? Organismal genome sizes span six orders of magnitude^1,2^ from ~160 kb (bacterial endosymbiont *Carsonella ruddii* ^3^) to ~150 Gb (angiosperm, *Paris japonica* ^4^). Genome size is a complex multivariate trait. Nevertheless, for viruses, prokaryotes and many unicellular eukaryotes, genome size can be explained largely by the gene number^2^. In addition to gene number, population genetic principles have been proposed to explain genome size and complexity^2,5^. The basic model is that organisms with smaller effective population sizes are less efficient at removing mildly deleterious mutations, in particular transposons and introns^2,6,7^. Thus, lineages with small population sizes will drift towards larger genomes. While the magnitude of the effect of population size on genome size variation is under debate, ranging from 8–64%^5^, the remaining variation in genome size is still largely unexplained.

In the Elegans subgroup of the *Caenorhabditis* genus, all 3 cases of the evolution of hermaphroditism have been associated with a convergent reduction in genome size relative to gonochoristic (male/female) species^8–13^. However, based on the effective population sizes in *Caenorhabditis* species, the genome sizes are opposite of the simple population size predictions. The gonochoristic species (*C. remanei* and *C. brenneri*) have larger population sizes than hermaphroditic species (*C. briggsae* and *C. tropicalis*), consistent with the expectations for outcrossing and selfing species^14–16^. However, gonochoristic species (*C. remanei*, *C. brenneri*, *C. japonica*, C*. sinica* and *C. nigoni*) have larger genome sizes than hermaphroditic species (*C. elegans*, *C. briggsae* and *C. tropicalis*)^8–10^.

Non-Mendelian chromosome assortment may also be associated with genome size differences in the *Caenorhabditis* genus. In males, there is preferential segregation of the longer chromosome of a heterozygous homologous pair away from the X chromosome and of the shorter chromosome with the X^17^. We call this phenomenon “skew”. Given that hermaphrodites tend to inherit the shorter chromosome and because new populations of *C. elegans* are frequently initiated by hermaphrodites^18–21^, skew may partially explain genome size reduction relative to the ancestral gonochoristic species.

If skew plays a role in genome size reduction within the clade, then skew should be present in other *Caenorhabditis* species. In this study, we tested this hypothesis by examining transgenic strains of multiple species across the *Caenorhabditis* genus and then observing chromosome inheritance patterns. We examined both integrated and extrachromosomal transgenes as they are compositionally similar in *Caenorhabditis* nematodes. Finally, we wanted to determine whether skew was male germline specific or general. We found that all eight of the tested species exhibited skew, suggesting that skew was probably present in the ancestral *Caenorhabditis* species.

## Results

### Integrated transgenes are transmitted preferentially from fathers to sons in both hermaphroditic and gonochoristic species

A previous study demonstrated skew in *C. elegans* ^17^. For comparison in this study, we retested an outcrossed *mIs10* strain by crossing heterozygous *mIs10/*+ males to Unc hermaphrodites. Counts of their nonUnc cross progeny confirmed skew with a transmission bias ratio (TBR; “preferred”/“anti” gamete combinations, see Methods) of 3.86. This value is smaller than previously observed for *mIs10* (TBR = 6.55^17^). The difference is likely because of one or more skew modifier mutations being removed or introduced during our outcrossing of *mIs10* (see Supplementary Text S1).

**Figure 1:**
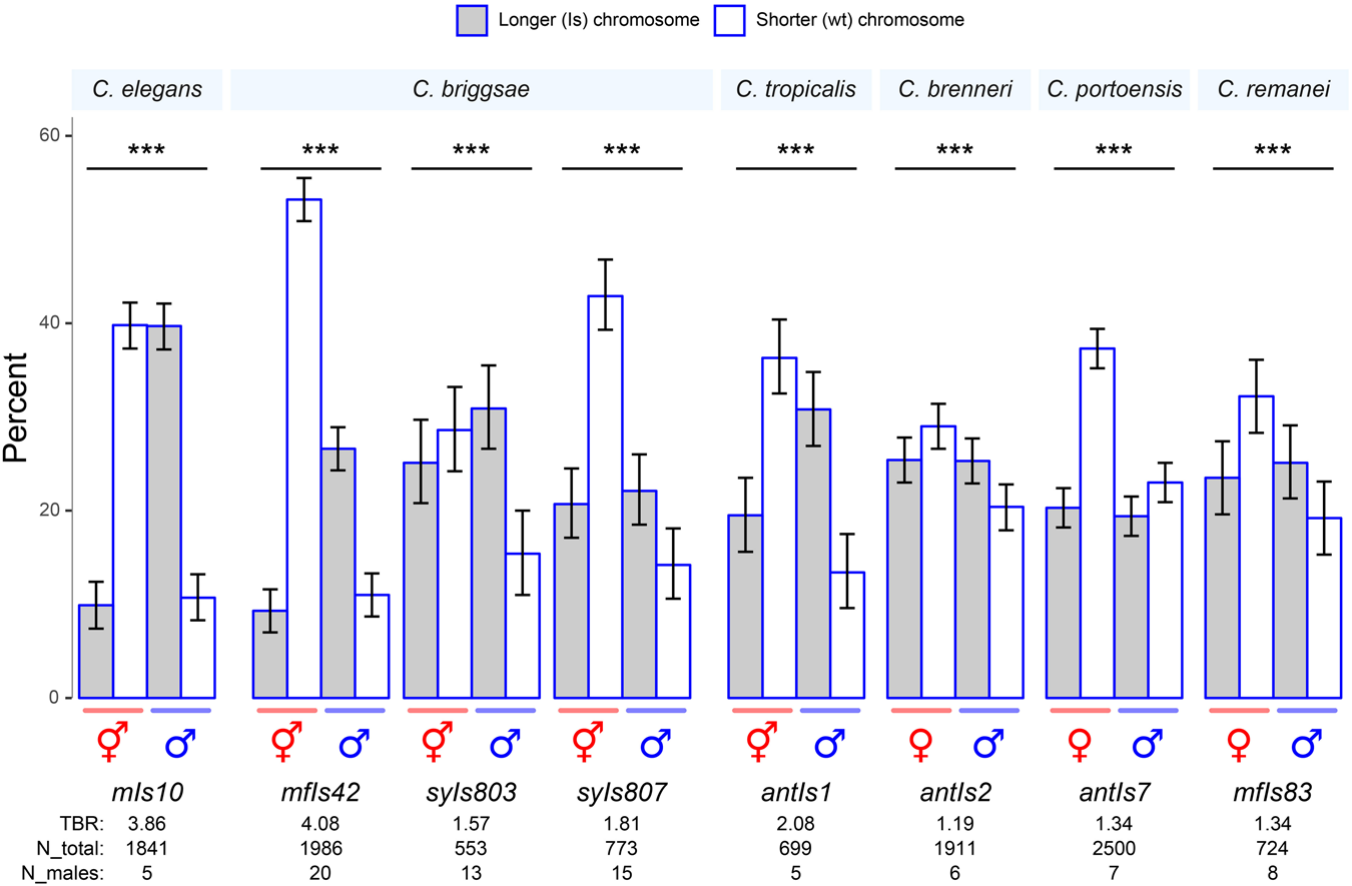
Transmission patterns of integrated transgenes. Percentages of individuals carrying integrated arrays (gray bars) or the wild-type chromosome allele (white bars). Species names are indicated above the plots. Transgene names are indicated below in italics. Error bars are 95% confidence intervals. *P-*values, χ^2^ tests assuming random segregation of the two alternate chromosomes by sex; ***P < 0.001. TBR, transmission bias ratio; N_total, total individuals scored; N_males, number of males tested.

Next, we tested the two other hermaphroditic species, *C. briggsae* and *C. tropicalis*, in a similar fashion. In *C. briggsae*, examination of three heterozygous integrated transgenes (*mfIs42*, *syIs803*, and *syIs807*) revealed skew in all cases with the TBRs ranging from 1.57 (*syIs803*) to 4.08 (*mfIs42*) (Fig. 1, all *P* < 0.001, χ^2^-test, df = 1). Likewise, *C. tropicalis* exhibited skew (*antIs1*, TBR 2.08; *P* < 0.001, χ^2^-test, df = 1).

To determine whether skew was present in gonochoristic species, we tested *C. brenneri* (*antIs2*), *C. portoensis* (*antIs7*), and *C. remanei* (*mfIs83* ^22^). In all three species, we observed skew (Fig. 1, TBR’s from 1.19 to 1.34, all *P* < 0.001, χ^2^-test, df = 1). Thus for all six species tested, we observed skew, regardless of the mode of reproduction employed, i.e., hermaphroditism or gonochorism.

TBR varied across the lines and species. In *C. elegans*, TBR is positively correlated with insertion size^17^. We tested for a similar correlation across six of the strains (four species) but found none (*P* = 0.41, Spearman’s rank correlation test) indicating that species-specific factors likely modify TBR. However, three *C. briggsae* strains show a suggestive pattern consistent with a positive correlation (Supplementary Figure S1). Examination of additional insertion strains within each species would clarify the magnitude of TBR explained by insertion length.

### Extrachromosomal transgenes were also transmitted preferentially from fathers to sons in hermaphroditic and gonochoristic species

**Figure 2:**
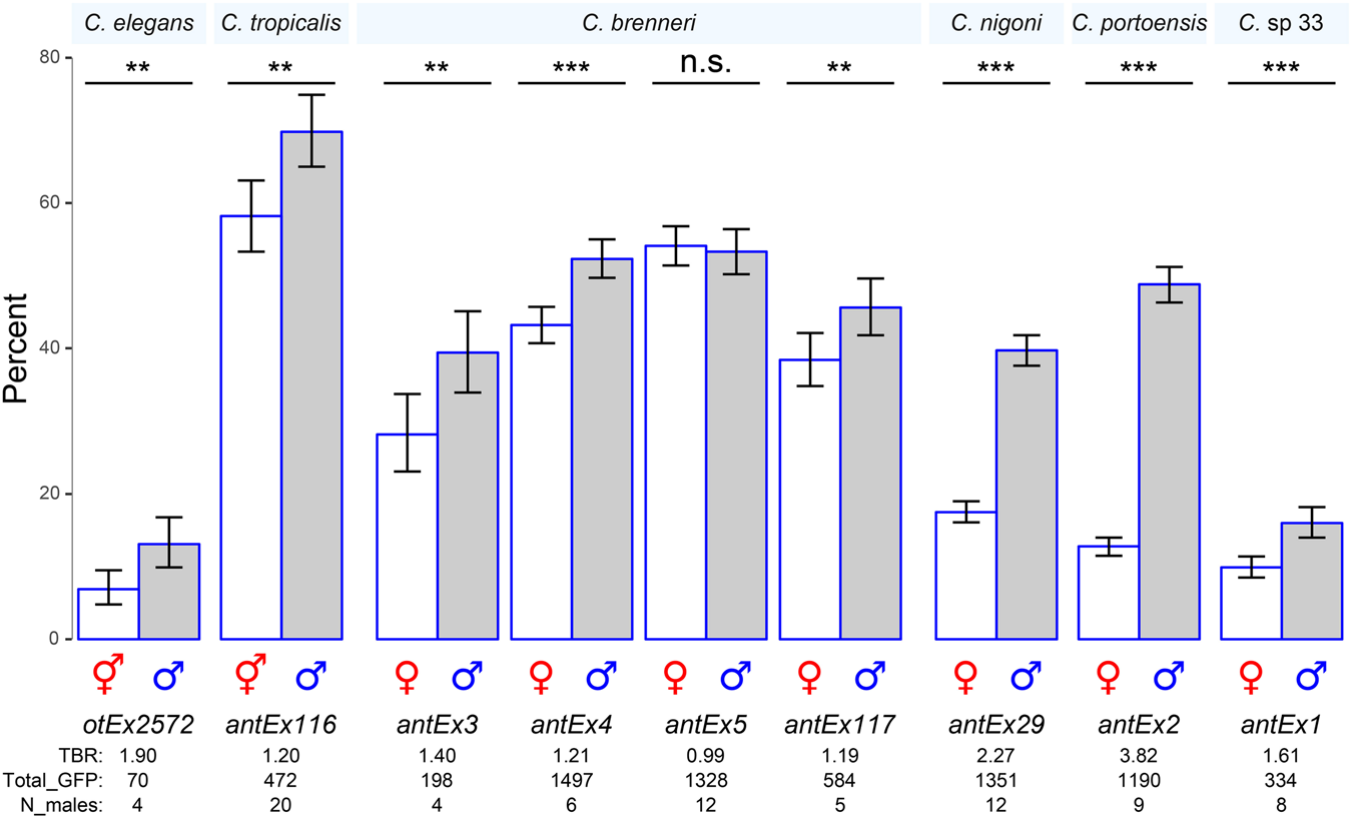
Transmission patterns of extrachromosomal transgenes. Percentages within each sex of hermaphrodites/females (white) and males (gray) inheriting the extrachromosomal array. Species names are indicated above the plots. Transgene names are indicated below in italics. Error bars are 95% confidence intervals. *P-*values, χ^2^ tests assuming random segregation of the extrachromosomal transgenes by sex; ***P < 0.001; **P < 0.01; n.s., not significant (*P* > 0.05). Note, the χ^2^ tests were conducted on the full progeny datasets (i.e., individuals by sex and inheriting or not inheriting the extrachromosomal transgene). TBR, transmission bias ratio; Total_GFP, total number of GFP individuals scored; N_males, number of males tested. See Supplementary Figure S2 for plots with all progeny.

Given the similarity of DNA composition between extrachromosomal and integrated arrays, we examined if the former also shows biased transmission from fathers to sons. We first tested two hermaphroditic species, *C. elegans* and *C. tropicalis*, by crossing males carrying extrachromosomal arrays with GFP or mCherry to tester hermaphrodites. These crosses revealed that extrachromosomal arrays were also preferentially transmitted to sons in both hermaphroditic species (both *P* < 0.008, χ^2^-test, df = 1, Fig. 2; and Supplementary Figures S2 and S3).

Next, we tested four gonochoristic species. For three species (*C. nigoni*, *C*. *portoensis*, and *C*. sp. 33), only one extrachromosomal line was generated, and in all cases we observed skew (all *P* < 0.001, χ^2^-test, df = 1; Fig. 2). We also observed skew in *C. brenneri* for three extrachromosomal lines (*antEx3, antEx4, antEx117*), but a fourth one did not (*antEx5*). The TBRs for these extrachromosomal arrays in these four species ranged from 0.99 to 3.82 (Fig. 2).

### Skew appears specific to the male germline

Thus far, all tests for skew assayed males which are XO and only produce sperm. In the following experiments, we tested if skew might be restricted to sperm or to males. Wild-type hermaphrodites also produce sperm, but since they are XX, testing for preferentially segregation of insertions away from the X is not possible. Thus, we used *fem-3* and *her-1* mutants where XO individuals are transformed into females (producing only oocytes) or self-fertile hermaphrodites, respectively.

**Figure 3:**
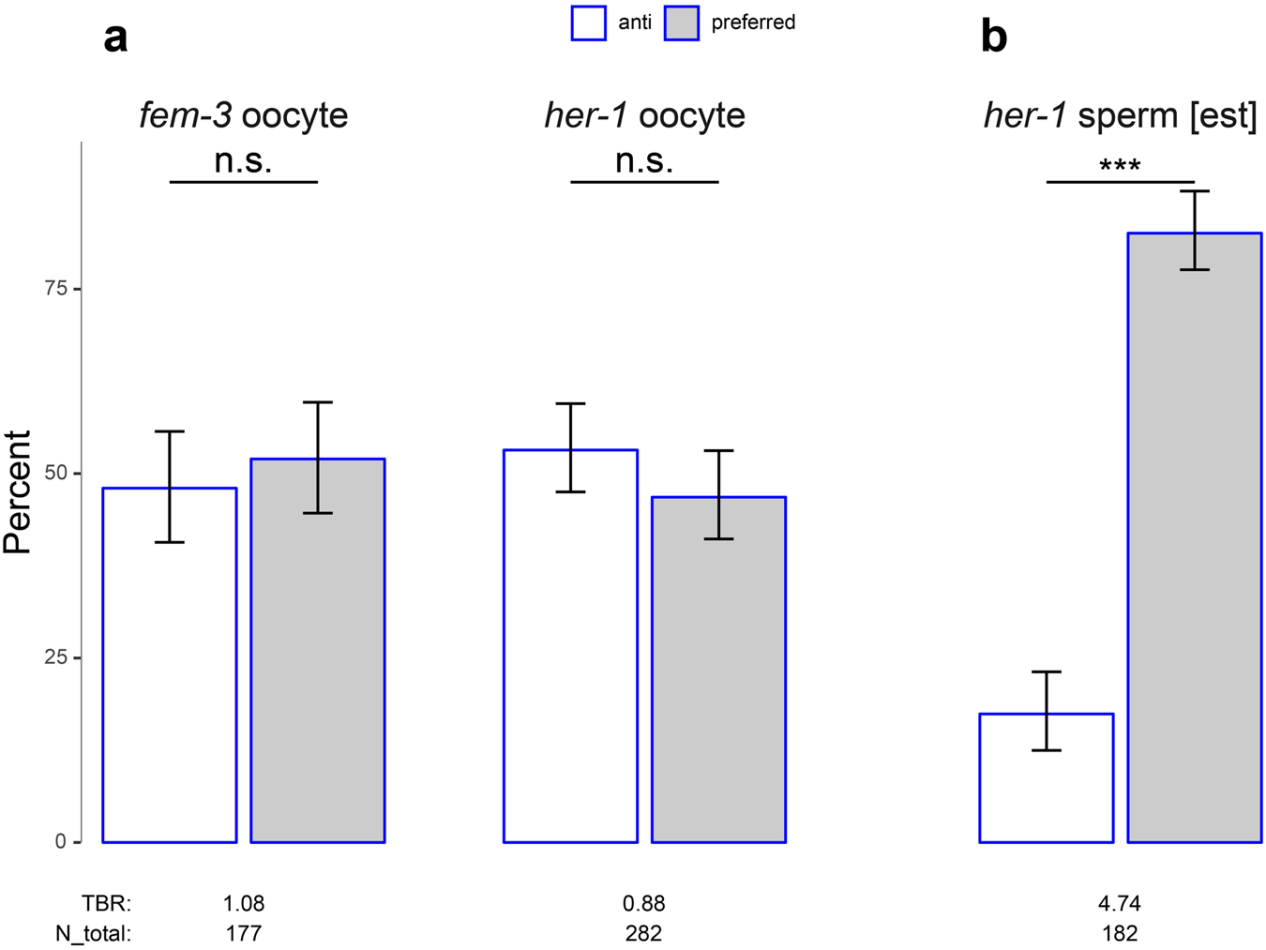
Transmission patterns of integrated transgenes in ooctyes and sperm. Percentages of progeny inheriting the “anti” (white) or “preferred” (gray) chromosome combinations (see). (**a)** Oocytes in *fem-3* and *her-1* mutants. *P*-values are from binomial tests assuming equal segregation of the two chromosome combinations (see also Supplementary Figure S4). (**b**) Sperm in *her-1* mutant. Percentages are estimated [est] from the observed ratio of 155:27::XO:XX individuals, and *P-*value is from a binomial test assuming 2:1 ratio of XO:XX individuals (see main text and Supplementary Figure S5 for details). Error bars, 95% confidence intervals. ***P < 0.001; n.s., not significant (*P* > 0.05); TBR, transmission bias ratio; N_total, total individuals scored.

We first tested whether oocytes exhibited skew by crossing *fem-3* XO pseudo-females heterozygous for a GFP transgene (*gfp/*+ *; fem/fem*) to males with an X-linked RFP (*rfp*/O) and scoring their progeny. The X-linked RFP transgene was necessary to identify sons that inherited the paternal X (RFP) as opposed to the maternal X (non-RFP). This cross yielded six viable offspring genotype classes (of eight possible) which we grouped into the “preferred” or “anti” gamete combinations (see Methods and Supplementary Figure S4). Of 177 individuals scored, 92 and 85 inherited the “preferred” and “anti” gamete combinations, respectively, which was not different from random assortment (Fig. 3A, *P* = 0.33, binomial test).

To confirm that this lack of skew in oocytes was not specific to the *fem-3* mutant background, we tested *her-1* mutants similarly. We crossed XO hermaphrodites (*her-1 mIs10*/*her-1* + V; XO) to *rfp*/O males and assayed the progeny phenotypes. Again, we found that oocytes did not exhibit skew (Fig. 3A; 132 preferred, 150 anti, *P* = 0.31, binomial test).

Finally, we tested if the sperm of XO *her-1* individuals (*her-1 mIs10*/*her-1* + V; XO) exhibited skew by looking for deviations from random segregation in self-progeny. Of the self-progeny the most discriminatory cases are the GFP/GFP homozygotes. If there is no skew in the hermaphrodite sperm (i.e., random segregation of chromosomes), then the predicted ratio of XO:XX individuals would be 2:1. In contrast, a transmission bias ratio of ~4:1 for *mIs10* (above) yields a predicted ratio of ~5:1 for XO:XX individuals (see Supplementary Figure S5). Examination of the self progeny revealed that the segregation pattern was not random (Fig. 3B; 155 XO: 27 XX; *P* = 2.3e-8, binomial test with likelihood of success = 2/3). This corresponds to a TBR of 4.74 for hermaphrodite sperm, which is not different from the ~4 TBR for *mIs10* in normal males (*P* = 0.55, binomial test with likelihood of success = 5/6). Together these data indicate that skew is not specific to the soma sex, per se, but is limited to male meiosis or some subsequent step of spermatogenesis.

## Discussion

**Figure 4:**
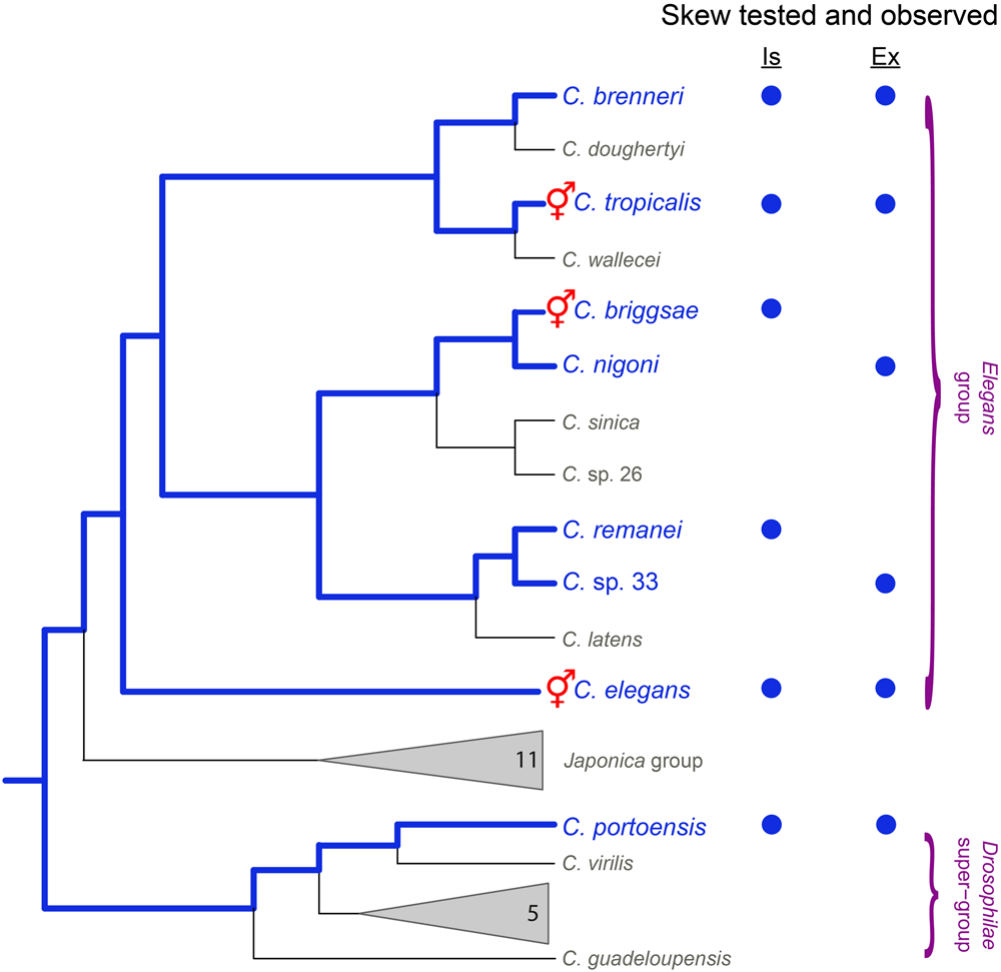
Skew across the *Caenorhabditis* genus. Summary of skew results placed on the *Caenorhabditis* phylogeny. All eight species tested exhibited skew for integrated (Is) arrays, extrachromosomal (Ex) arrays, or both (blue names and circles). Blue lines indicate inferred ancestral, evolutionary lineages with skew. Other species and clades within the *Caenorhabditis* are likely to have skew but are untested (gray text and triangles; numbers indicate number of known species within). Phylogeny adapted from^12,70^ with the position of *C*. sp 33 placed next to *C. remanei* based on ITS2 sequence (unpublished data).

We found that the transmission of transgenes preferentially toward sons is present in all *Caenorhabditi*s species tested. Skew occurs in *C. elegans* whereby fathers transmit autosomes that are heterozygous in length to their offspring in a non-Mendelian fashion^17^. In this study, we used integrated transgenes to demonstrate that all three known hermaphroditic species and three gonochoristic species of *Caenorhabditis* exhibit skew (Fig. 1). We also showed that extrachromosomal arrays are preferentially transmitted from fathers to sons rather than daughters in six species (Fig. 2). Because of the similar transmission patterns and DNA composition for both integrated and extrachromosomal transgenes, we refer to this transmission bias in both as skew. In total, we observed skew in all eight of the *Caenorhabditis* species tested (Fig. 4).

To our knowledge, this study is the first to systematically examine skew in multiple species in the *Caenorhabditis* genus. However, skew or likely cases of it have been noticed previously. Several early *C. elegans* studies observed that free duplications of partial chromosomes were transmitted preferentially from fathers to sons in *C. elegans* ^23–26^. More recently, a study examining hybrid incompatibility found that *C. nigoni* males heterozygous for a *C. briggsae* introgression (strain ZZY10028) preferentially transmitted the *C. nigoni* orthologous genomic fragment to sons^27^. As the *C. nigoni* genome is larger than that of *C. briggsae* ^28^, the focal homologous genomic fragment is presumably longer than the introgressed *C. briggsae* counterpart, and thus the chromosome transmission patterns are consistent with skew^27^.

With the current evidence, it is very likely that all the species in the Elegans subclade display skew. Our study also included one species, *C. portoensis*, that is outside of the Elegans subclade. This would suggest that skew is conserved among all *Caenorhabditis* species and was likely already present in the ancestor of the *Caenorhabditis* genus.

The finding that all *Caenorhabditis* species have skew is important because this observation supports the possibility that smaller genomes in hermaphroditic (all circa 100 Mbp) compared to gonochoristic species (all circa 130 Mbp)^9,10,13^ could be due, in part, to skew.

How might skew have led to genome reduction in hermaphroditic species? With skew, every time a male carrying a heterozygous deletion (or insertion) mates to a hermaphrodite, the daughter hermaphrodites will tend to inherit the shorter allele. Because a few or even a single hermaphrodite individual is sufficient to colonize a patch, an opportunity for a ratchet-like reduction in genome size could occur after every such mating involving a deletion. In contrast, for gonochoristic species although daughters would preferentially inherit the shorter chromosomes, sons would preferentially inherit the longer one. On average, matings should mix the slightly smaller female genomes with the slightly longer male ones resulting in no directional change in genome size. This model is supported by simulations^17^. Thus, the current *Caenorhabditis* genome size patterns could partially reflect skew operating over evolutionary time scales.

Reduction of hermaphrodite genome sizes due to skew requires mating. Direct observations of males in hermaphroditic species are rare^18,19,21^ and genetic estimates of the natural mating rate in extant hermaphroditic species is low^18,19,29^. However, matings were likely more frequent at the onset of hermaphrodite evolution, potentially implying that the rate of genome size reduction may have been faster at that time. The evolution of hermaphroditism can occur with mutations in only two genes^30,31^, which do not cause reproductive isolation. Thus, in any potential cases of abrupt evolution of hermaphroditism, matings may still occur frequently with sympatric males of the originating gonochoristic sister species before speciation has occurred. A more gradual evolution of hermaphroditism, perhaps via an intermediate mating system with males, females, and hermaphrodites (e.g., the free-living Rhabditis sp. SB347^32–34^ and the entomopathogenic *Heterorhabditis* species^35,36^), would imply more mating occurrences, and again more opportunity for genome reduction.

While skew could cause genome reduction, whether it has is unclear. Genome size can be affected by many factors including directional bias in mutations, effective population size, and transposable element accumulation^37–39^. A recent study comparing the genomes of the three hermaphroditic species with five gonochoristic species came to the conclusion that the main driver for hermaphrodite genome reduction was probably the loss of genes associated with traits no longer needed^9^. Some gene losses may have been beneficial because the associated mating or sex-specific traits were costly to maintain; more investment could be made in other aspects of its biology. Other, and possibly most, genes and traits were probably lost because of relaxed selection. Genome reduction in this model would not require mating, and hence, would not require skew.

Nevertheless, skew is still compatible with both positive and relaxed selection because any deletion would need to spread throughout the population in order to reach fixation. In principle, a deletion strain experiencing positive selection could completely sweep through the population in a clonal fashion. With matings the spread of a beneficial deletion allele would be accelerated because the direction of selection and skew are coincident. During relaxed selection, drift would play a stronger role than selection, and for truly neutral deletion alleles fixation or loss is proportional to its allele frequency. In these cases, skew could be a driver, providing directionality for the fixation of neutral or nearly neutral deletion alleles.

Quantifying the contribution, if any, of skew on genome size reduction in hermaphroditic species may be challenging. One possibility would entail long-term experimental evolution that contrasted an obligately mating strain (e.g., *fog-2* or *spe-27* mutants^40,41^) to a hermaphroditic strain experiencing episodic matings. Genome comparisons between true sister species pairs of hermaphroditic and gonochoristic species may also be enlightening.

In addition to a potential impact on genome size differences, skew may have an impact on sexually antagonistic alleles^42^, which are alleles that benefit one sex but harm the other. When an allele is found equally often in males and females (as for typical autosomal genes), the allele’s beneficial or harmful effects often need to be balanced between the sexes. However, if an allele is more frequently found in one sex, then sexual antagonism may be easier to evolve. The sex chromosomes in heterogametic organisms (e.g., X and Y chromosomes) are an extreme example. Given skew in *Caenorhabditis* species, selection may be possible for male beneficial (and female detrimental) alleles on the longer chromosomes and female beneficial (and male detrimental) alleles on the shorter chromosomes^42^. As there is natural variation in gene copy number in *C. elegans* and other nematodes^43–46^, it is likely that wild variation in copy numbers of genes is common. Thus, an interesting future avenue will be to test if there are any such sexually antagonistic alleles, especially in the gonochoristic species where the two sexes are always present.

Meiotic cells can segregate chromosomes that are not joined by chiasmata (sites of crossovers during homologous recombination) through a process called achiasmate segregation. Achiasmate segregation is often considered a “backup” mechanism to segregate homologous chromosomes that fail to form crossovers. This is also the primary mechanism to segregate some autosomes that never form crossovers such as the 4th chromosome in females^47^ and all chromosomes in males in *Drosophila melanogaster*. Achiasmate segregation of heterologous chromosomes also occurs, for example for the heterogametic sex chromosomes that do not have shared regions of homology (i.e., lack pseudoautosomal regions)^48^, compound chromosomes in *D. melanogaster* ^49,50^, and artificial chromosome pairs in yeast^51^. Skew in *C. elegans* is also a form of heterologous achiasmate segregation (i.e., the solitary X and a longer autosome, which itself is synapsed with its shorter homolog). Achiasmate segregation has also been observed in *C. elegans* for both free duplications^23–26^ and autosomes^52^.

In the *C.elegans* autosome case, homologous achiasmatic segregation appears to occur only in sperm and not in oocytes^52^. Our experiments revealed a similar difference for heterologous achiasmatic segregation between the gametes for skew. During spermatocyte meiosis, an integrated transgene preferentially segregated away from the X, while during oocyte meiosis (in *fem-3* or *her-1* XO animals) it segregated randomly with respect to the solitary X. The difference in achiasmate segregation between spermatocyte and oocyte meiosis could be due to the presence of centrioles in spermatocytes and their absence in oocytes^53^, differences in the organization of the meiotic spindles^54,55^, accelerated meiotic progression kinetics in spermatocytes^54^, distinct morphologies of the meiotic chromosomes^54^, the presence of a solitary X in males, and/or other unknown differences. From an adaptive perspective, sperm-specific achiasmate segregation may imply that *Caenorhabditis* meiosis is more prone to failure to form chiasmata in spermatocytes than oocytes.

Finally, during the course of this study, we noticed that all seven non-*C. elegans* species produced at least one cross with female-biased sex ratios (11 crosses with *P* < 0.0029; Bonferroni adjusted threshold; binomial test; Fig. 1 and Supplementary Figure S2). Sperm competition or temporally-delayed usage of O-bearing sperm has been implicated previously for *C. briggsae* ^56^ and a similar mechanism may explain the observed pattern.

In summary, we report that skew is likely the ancestral state in *Caenorhabditis* species, which may contribute to the patterns of genome size in this genus. We also show that skew is specific to spermatocyte meiosis. Finally, depending on the extent of conservation of the meiotic machinery, it is possible that skew extends to other nematode taxa.

## Materials and Methods

### Nematode culture and *Caenorhabditis* strains

All strains were maintained on Nematode Growth Medium (NGM) plates seeded with *E. coli* OP50 at 20°C in a TKS Low-Temp incubator LTI 603 or at room temperature (~22°C). The following strains were obtained from the *Caenorhabditis* Genetics Center (CGC) or generated in this study. Strains are ordered as follows: wild-type, mutants, transgenics.

*C. elegans*: N2 var. Bristol (*wild-type* (*wt*)), BRC174 (*unc-24(e138) fem-3(e1996)/unc-24(e138) dpy-20(e1282) IV*), BRC183 (*unc-24(e138) dpy-20(e1282) IV*), BRC189 (*unc-119*(*ed9*) *III; ttTi5605 II* [EG4322 out-crossed 10 times with N2]), CB3843 (*fem-3*(*e1996*)/*unc-24*(*e138*) *dpy-20*(*e1282*) IV), DR108 (*dpy-11*(*e224*) *unc-42*(*e270*) *V)*, XA8150 (*dpy-11(e224) her-1(e1518) V*), BRC148 (*unc-119*(*ed3*) *III; him-8(e1489) IV; antEx50*[*Puro-R, Neo-R, myo-2::GFP, sur-5::GFP, myo-3::mCherry*]), BRC153 (*unc-119*(*ed3*) *III; him-8(e1489) IV; antEx51*[*Puro-R, Neo-R, myo-2::GFP, sur-5::GFP, myo-3::mCherry*]), BRC237 (*unc-24(e138) fem-3(e1996)/unc-24(e138) dpy-20(e1282) IV; mIs10 V*), OH4460 (*otEx2572*[*unc-97::NLS::GFP*]), PD4793 (*mIs10*[*myo-2::GFP*, *pes-10::GFP*, *F22B7.9::GFP*] *V*), WHR10 (*mIs10 V*; [*mIs10* in PD4793 was out-crossed 10 times with N2]), and XA8160 (*dpy-11(e224) her-1(e1518) mIs10 V*).

*C. briggsae*: AF16 (*wt*), BC1983 (*Cbr-dpy*(*s1281*)), JU929 (*Cbr-dpy-18* (*mf104*) *III*), JU1018 (*mfIs42*[*Ce-sid-2*, *Ce-myo-2::*DsRed])^22^, PS9392 (*syIs803*[*Ce-daf-4*( + ), *myo-2::*GFP] *II*)^57^ and PS9396 (*syIs807*[*Ce-daf-4* ( + ), *myo-2::*GFP] *IV*)^57^.

*C. brenneri*: CB5161(*wt*), JU1397(*wt*), BRC467 (*antIs2*[*myo-2::*GFP, *sur-5::*GFP]), BRC532 (*antEx3*[*myo-2::*GFP, *sur-5::*GFP]), BRC536 (*antEx117*[*myo-2::*GFP, *sur-5::*GFP]), BRC567 (*antEx5*[*myo-2::*GFP, *sur-5::*GFP]), and BRC579 (*antEx4*[*myo-2::*GFP, *sur-5::*GFP]).

*C. remanei*: BRC20108 (*wt*, isolate from Okinawa), PB4641 (*wt*), and BRC534 (*mfIs83*[*Cel-sid-2* + *Cel-myo-2::*DsRed]; allele previously known as *mfEx34*, M.-A. Félix personal communication) (*mfIs83* in JU1184 was out-crossed 8 times with BRC20108)^22^.

*C. tropicalis:* JU1373 (*wt*), BRC461 (*Ctr-dpy(ant27)*), BRC493 (*antEx116*[*myo-2::mCherry*]), and BRC555 (*antIs1*[*myo-2::mCherry*]).

*C. portoensis*: EG4788 (*wt*), BRC313 (*antEx2*[*myo-2::*GFP]) and BRC585 (*antIs7*[*myo-2::*GFP]).

*C. nigoni*: BRC10094 (*wt*, isolate from Taiwan) and BRC340 (*antEx29*[*myo-2::*GFP, *sur-5::*GFP]).

*C*. sp. 33: BRC10016 (*wt*, isolate from Taiwan) and BRC311 (*antEx1*[*myo-2::*GFP]).

### Generating transgenic strains

All plasmids (pdestDD04neo and pDD04neo^58^, pBCN21-R4R3^59^, pPD158.87^60^, pCFJ151 and pCFJ104^61^) were prepared for injection using standard protocols. In brief, each plasmid was transformed into DH5α *E. coli* cells. Plasmid containing bacteria were cultured in LB broth containing 50 ng/ml ampicillin overnight at 37°C and then purified with the QIAprep Spin Miniprep kit (Qiagen) with final elution using 50 µl of distilled water.

To make transgenic strains for the hermaphroditic species, *C. elegans* (N2) and *C. tropicalis* (JU1373), we established extrachromosomal lines using standard *C. elegans* protocols (Supplementary Table S1)^62–64^.

For the gonochoristic *Caenorhabditis* species (*C. brenneri* (CB5161)*, C. portoensis* (EG4788)*, C. nigoni* (BRC10094), and *C*. sp 33 (BRC10016)), we adapted the microinjection methods for *C. elegans* ^62–64^ and *C. remanei* ^22^ as follows. We used a Narishige IM300 microinjection system attached to a Zeiss Vert A1 microscope to inject the plasmid mixture into the distal arms of the anterior and posterior gonads of virgin wild-type (WT) young adult females (females were isolated at the L4 stage). Every injected female was placed individually with three to four WT L4 males on an OP50-seeded NGM plate. The F1 progeny were examined daily under an epifluorescent microscope for individuals that expressed one or more of the injected transgenic markers. Specifically, we looked for green fluorescent protein (GFP) or mCherry in the pharynx driven by the *Cel*-*myo-2* promotor or in the hypodermis and intestine driven by the *Cel*-*sur-5* promotor (pPD158.87). Subsequently, single L4 transgenic F1 individuals were placed with three WT L4 individuals (except for *C*. sp. 33, see below) of the opposite sex.

We enriched for transgenic individuals by selection on antibiotic NGM plates. We prepared NGM plates containing 0.35 mg/ml G418 (Sigma-Aldrich, A1720-25G) seeded with 100 µl of OP50 that had been cultured in LB broth overnight and concentrated by centrifugation^58^. Single transgenic F2 individuals were crossed to three to five WT individuals of the opposite sex. This was repeated until we were confident that the transgenic line was established, which in all cases was at least 7 generations.

The *myo-2*::GFP signal was hard to detect at the L3, L4 and adult stages for *C*. sp. 33. Thus, each L1 or L2 transgenic F2 larva was transferred to a separate NGM plate. Upon reaching the L4 stage, two WT L4 individuals of the opposite sex were mated to each transgenic worm. Subsequently, matings between brothers and sisters were conducted for multiple generations until the transgenic line was established.

### Generating integration lines

Integrated lines were already available for *C. elegans* and *C. briggsae*. In addition, *mfEx34* in *C. remanei* appears to be a spontaneously integrated transgene^22^ (and our observations); thus this allele has been renamed *mfIs83* (see above). We generated integrated lines for three additional species: *C. brenneri, C. portoensis*, and *C. tropicalis*.

For *C. brenneri*, several lines appeared to have very high transmission rates of the extrachromosomal transgenes after culture on G418-containing NGM plates, possibly indicative of spontaneous integration. To identify integrants, we selected four to five transgenic gravid females (P0) from each putatively integrated line to separate NGM plates. From the P0 families that produced all or nearly all GFP F1 progeny, we crossed single L4 transgenic F1 males to single L4 transgenic F1 females on NGM plates without G418 and found two strains with 100% transmission of GFP in the F2 generation, suggesting that they likely carried spontaneously integrated transgenes. We conducted a second test to verify integration in these two apparently 100% transmission lines. If transmission is due to homozygosity rather than exceptionally high transmission of an extrachromosomal array, then crossing to WT should yield a heterozygous *gfp/*+ individual that should bear ~50% GFP cross progeny when crossed again to WT individuals. One strain fulfilled this criteria, and we renamed the transgene *antIs2*.

For *C. portoensis*, approximately 30 L4 extrachromosomal array carrying females (BRC313 (*antEx2*[*myo-2::*GFP])) were irradiated using a Spectroline UV crosslinker at a power setting of 250 (×100 µJ/cm^2^). Single irradiated P0 females were transferred to *E. coli*-seeded NGM plates and crossed to five WT L4 males. Each F1 GFP individual was crossed to two WT L4 individuals of the opposite sex. If the array was integrated onto an autosome (presumably as a heterozygote), then the predicted GFP:non-GFP ratio in the F2 generation would be approximately 50:50. For such broods, single F2 GFP males were crossed to single F2 GFP females (both putatively *gfp/*+). We then selected the F2 families producing approximately 75% GFP (both *gfp*/*gfp* and *gfp/*+) and 25% non-GFP progeny (+*/*+) in the F3 generation. To eliminate potential mutations caused by the integration procedure, we out-crossed single transgenic individuals (*gfp*/*gfp* or *gfp/*+) to two WT worms of the opposite sex. This was repeated for six generations. Next, we crossed (inbred) single L4 GFP brothers to single L4 GFP sisters repeatedly for three to four generations. In the end, we obtained one homozygous line (100% GFP progeny; BRC585 (*antIs7)*).

For *C. tropicalis*, approximately 60 L4 extrachromosomal array carrying hermaphrodites (BRC493 (*antEx116*[*myo-2::mCherry*])) were UV-irradiated as above, and then two to five irradiated P0 individuals were transferred to *E. coli*-seeded NGM plates. Subsequently, F1 mCherry individuals (n = 209) were singled to new plates. The F2 progeny of potential F1’s with heterozygous integrants should segregate approximately 75% mCherry, and from these candidate plates, two to four F2 potentially homozygous mCherry individuals were singled to new plates. Picking of candidate homozygous mCherry individuals was repeated in the F3 and subsequent generations, resulting in one homozygous line. This strain was then outcrossed to WT (JU1373) three times to obtain BRC555 (*antIs1*). While outcrossing, the integration status of *antIs1* was confirmed by candidate heterozygous males producing approximately 50% mCherry cross progeny, in contrast to a higher rate of transmission indicative of an extrachromosomal array.

### Generating mutants for *C. tropicalis*

To aid assessing cross progeny during crosses with the hermaphroditic species *C. tropicalis*, we conducted a standard F2 EMS screen in *C. tropicalis* (JU1373) for strains with clear recessive morphological mutant phenotypes. After screening ~2,000 genomes, we isolated five *Ctr-dpy* (*ant23, 25, 26, 27, 32*), four *Ctr-unc* (*ant20, 22, 24, 28*), one *Ctr-egl* (*ant29*), and one *Ctr-rol* (*ant21*) mutants with 100% penetrance and strong phenotypes. We outcrossed some of these strains 2-3 times back to JU1373 to remove mutations before choosing the 3x outcrossed *Ctr-dpy* (*ant27*) strain BRC461 for testing cross progeny. Backcrossing revealed that *Ctr-unc*’s (*ant20, 28*) and *Ctr-dpy (ant32)* are on the X-chromosome.

### Testing for skew of integrated transgenes

For the hermaphroditic species, single homozygous transgene (e.g., *gfp/gfp*) L4 males were crossed to WT L4 hermaphrodites. Their heterozygous F1 L4 males were crossed singly to two or three L4 Dpy or Unc hermaphrodite testers. Each set of parents was transferred together to new OP50-seeded NGM plates every day until they stopped producing F2 cross progeny. All F2 cross progeny were counted for transgenic (Green/GFP or Red/mCherry) and non-transgenic (non-Green or non-Red/mCherry) females and males at the L4 or adult stage; the Dpy or Unc self progeny were not counted. We used the following mutants for testing: *unc-119*(*ed3*), *C. elegans*; *Cbr-dpy*(*s1281*) or *Cbr-dpy-18*(*mf104)III, C. briggsae*; and *Ctr-dpy*(*ant27*), *C. tropicalis*.

For the gonochoristic species (*C. remanei*, *C. brenneri*, and *C. portoensis*), we tested heterozygous F1 L4 males using WT virgin female testers.

### Testing sex-biased transmission of extrachromosomal transgenes

For the *C. elegans* strain OH4460 (*otEx2572*), we crossed single transgene carrying males to two or three *dpy-11 unc-42* mutant hermaphrodites and then scored all nonDpyUnc individuals for transgenic (Green) and non-transgenic (non-Green) females and males at the L4 or young adult stage. For two other *C. elegans* strains, BRC148 (*unc-119(ed3)*; *antEx50*) and BRC153 (*unc-119(ed3); antEx51*), we crossed transgene carrying males to three *unc-119*(*ed3*) hermaphrodites. The F1 cross progeny inheriting *antEx50* or *antEx51* carry *Cb-unc-119* (+), and thus, were non-Unc. The numbers of non-Unc males to non-Unc hermaphrodites in the F1 generation were counted. We did not consider Unc progeny because we could not distinguish between self and cross progeny.

For the gonochoristic species, we tested *C. brenneri*, *C. nigoni, C. portoensis*, and *C*. sp. 33. Because many of the transgenic lines had poor fecundity, possibly due to inbreeding of the original WT strain, we selected the healthier transgenic lineages. We crossed single P0 males carrying an extrachromosomal array to WT females. Next, we crossed single F1 transgenic males to one or two WT virgin female testers. All F2 progeny at the L4 or adult stage were counted for transgenic (Green/GFP) and non-transgenic (non-Green/non-GFP) females and males.

All of the transgenic *C. brenneri* strains were extremely unhealthy (brood sizes often <20), and thus, we decided to backcross to a second wild isolate JU1397. We backcrossed each transgenic line seven to 12 times resulting in strains that produced larger broods (Supplementary Table S1). We used these strains to test for skew.

### Testing for skew in *fem-3* and *her-1* oocytes

For the *fem-3* oocyte skew experiments, the test individuals were pseudo-females with the following genotype: *unc-24 fem-3 IV*; *mIs10/*+ *V; XO*. To simplify obtaining individuals of this genotype, we chose to construct two intermediate strains (BRC174 and BRC237) as follows. First, from strain CB3843 (+*fem-3*+/*unc-24* + *dpy-20 IV*), we picked F1 Unc-nonDpy individuals. We then identified and kept an *unc-24 fem-3*+/*unc-24* + *dpy-20* F1 (strain BRC174) based on it being Unc and segregating three classes of individuals: fertile Unc, sterile Unc pseudo-females (seen as an accumulation of oocytes in gonads), and fertile UncDpy. Next, we crossed an F2 *unc-24 fem-3* pseudo-female to *mIs10* (*gfp V*) males. The F3 cross progeny males (*unc-24 fem-3/*++ ; *mIs10/*+) were then crossed to *unc-24 dpy-20* hermaphrodites to yield F4 *unc-24 fem-3*+*/unc-24* + *dpy-20*; *mIs10/*+ individuals based on having the Unc-nonDpy GFP phenotype. Subsequently, bright GFP Unc F5 individuals were picked and the *unc-24 fem-3*+*/unc-24* + *dpy-20 IV; mIs10 V* (BRC237) genotype was verified by broods consisting of three different classes of GFP progeny: fertile Unc, sterile Unc, and fertile Unc Dpy.

After obtaining BRC174 and BRC237, we set up the following crosses to assess skew in *fem-3* oocytes. We crossed N2 males to *unc-24 fem-3 IV*; *mIs10 V* pseudo-females (derived from BRC237), and then crossed the heterozygous F1 males to *unc-24 fem-3* pseudo-females (derived from BRC174). The desired F2 genotype was *unc-24 fem-3 IV*; *mIs10/*+ *; XO*, so we picked many Unc GFP L4 putative pseudo-females to individual plates and let them grow overnight in isolation. Rare Unc nonFem (*unc-24 fem-3/unc-24*+) recombinant hermaphrodites were excluded because they would be self fertile. The remaining pseudo-females, which were either XX or XO, were crossed to *rfp/O* males (*wyIs57*). XX mothers could be identified because they had large brood sizes and also because they never produced RFP males. In contrast, XO individuals had low broods (partly due to inviable OO progeny), many males (approximately 2/3), and some RFP males.

For *her-1*, we used a similar strategy. First we built strains XA8150 (*dpy-11 her-1* V) and XA8160 (*dpy-11 her-1 mIs10 V*). Then, to test for oocyte skew, we crossed N2 males to *dpy-11 her-1 mIs10* hermaphrodites. Next, F1 males were crossed to *dpy-11 her-1* hermaphrodites. This cross yielded three genotypes of hermaphrodites: 1) the desired *dpy-11 her-1 mIs10/*+ *; XO, 2) dpy-11 her-1 mIs10/*+ *; XX*, and 3) rare recombinant Dpy nonHer *dpy-11 her-1/dpy-11* + *; XX*. As above, we set up many crosses of one Dpy GFP L4 hermaphrodite and one *rfp/O* male (*wyIs57*). This cross would reveal XX mothers (both type 2 and 3) because they had large brood sizes and also because they never produced RFP males; such crosses were discarded. *her-1 XO* individuals were identified based on low broods (partly due to inviable OO progeny), many males (approximately 2/3), and some RFP males.

For both the *fem-3* and *her-1* experiments, we scored the sex, GFP, and RFP phenotypes from the crosses to XO mothers.

### Testing for skew in *her-1* sperm

We selfed the *dpy-11 her-1 mIs10/*+ hermaphrodites (above) and scored the brood sizes of single, cloned, strong GFP individuals, which were likely *gfp/gfp*. Of the self-progeny, the most discriminatory cases are the *gfp/gfp* homozygotes, which can be determined by intensity for *mIs10* (see Supplementary Figure S5). We further verified that all progeny (when present) expressed bright GFP. We used brood size to distinguish XX and XO individuals. XX have many progeny whereas XO have none or few. In this study, there was a bimodal pattern of brood sizes with all broods having either >40 or < 30 progeny, which we scored as XX and XO, respectively.

If the *mIs10* transgene segregated randomly, then the expected ratio of XO to XX progeny would be 2:1. Alternatively, if *mIs10* segregated away from the X at approximately the same rate as for normal males (~4:1 for nullo-X::X), then the expected ratio of progeny would be ~5:1 (see Supplementary Figure S5). Thus, we conducted binomial tests for departure from these ratios.

### Transmission bias ratio (TBR)

We defined the TBR as the ratio of the sex-normalized “preferred” to “anti” gamete combinations. “Preferred” was 1) wt chromosome segregating with X and 2) transgene chromosome segregating with nullo-X; “anti” was 1) transgene chromosome segregating with X and 2) wt chromosome segregating with nullo-X. For crosses with integrated arrays, “preferred” corresponded to non-transgenic daughters and transgenic sons while “anti” corresponded to transgenic daughters and non-transgenic sons. For the *fem-3* and *her-1* oocyte crosses, there were six viable offspring genotype classes (of eight possible) of which three corresponded to the “preferred” gamete combinations and three to the “anti” (see Supplementary Figure S4). For crosses with extrachromosomal arrays, “preferred” was extrachromosomal transgene segregating with nullo-X (i.e., transgenic sons) and “anti” was extrachromosomal transgene segregating with X (i.e., transgenic daughters). Non-transgenic progeny were excluded in the calculation because not inheriting an extrachromosomal array could be due to either standard chromosome segregation or spontaneous loss. Because some species have female-biased sex ratios, we normalized gamete combinations to its percent within each respective sex.

### Insertion size and transmission bias

For the comparison between WHR10 and PD4793, we estimated insertion sizes as previously described^17^ (Supplementary Table S2). For the interspecies comparisons, we could not use the same set of reference primers for DNA qPCR due to sequence divergence among the species. Therefore, we identified a putative highly conserved element, LG2_6028, using the iHCE software package^65^ (Supplementary Text S2 and Supplementary Table S2). We then conducted genomic DNA qPCR assays on *bla* ^66^ (plasmid marker) and LG2_6028 for six strains from four species (*C. elegans, C. briggsae, C. tropicalis*, and *C. brenneri*). Additional details are in Supplementary Text S2.

### Statistical analysis

Statistical analyses were conducted in R (version 3.4.0)^67^. Some figures were plotted using ggplot2^68^. For the skew tests in which both sex and transgene status were scored, we used the Chi-squared test (chisq.test function in R) with the null hypothesis being equal segregation of transgenes between hermaphrodites (or females) and males (i.e., 50% in both sexes for integrated arrays and a constant but equal number for extrachromosomal arrays). For the *fem-3* oocyte, *her-1* oocyte, and two extrachromosomal line crosses, we used the binomial test (binom.test function in R) to test for departure from equality between the preferred and anti categories. For *her-1* sperm, we tested against departure from expected progeny ratios with (5:1) and without skew (2:1, see above). Confidence intervals were calculated using the multinomialCI function^69^ in R. For the correlation tests between the TBRs and estimated insertion sizes (after adjusting for genome size) we used the Spearman’s rank correlation test.

## Electronic supplementary material

Supplementary Information
