# Supplementary material for: Non-Mendelian assortment of homologous autosomes of different sizes in males is the ancestral state in the Caenorhabditis lineage
Source: Sci Rep. 2017 Oct 9;7:12819. doi: 10.1038/s41598-017-13215-4 (PMC5634442; doi:10.1038/s41598-017-13215-4)
Supplement: Supplementary file 1 — Supplementary Information [file 41598_2017_13215_MOESM1_ESM.pdf]

Supplementary Information for:

Non-Mendelian assortment of homologous autosomes of different sizes in males is the ancestral state in the *Caenorhabditis* lineage

Tho Son Le[§†], Fang-Jung Yang[§], Yun-Hua Lo[§], Tiffany C. Chang[§], Jung-Chen Hsu[§], Chia-Yi Kao[§], John Wang[§*]

[§]Biodiversity Research Center, Academia Sinica, Taipei 11529, Taiwan

[†]Department of Molecular Genetics and Gene Technology, College of Forestry Biotechnology, Vietnam National University of Forestry, Hanoi, Vietnam

[*]Corresponding author

Email: johnwang@gate.sinica.edu.tw

This PDF file includes:

Supplementary Texts S1 and S2

Supplementary Figures S1 to S5

Supplementary Tables S1 and S2

Supplementary References

Supplementary Text S1. The skew transmission bias ratio difference for *mIs10* between the WHR10 and PD4793 strains is likely due to genetic modifiers.

The transmission bias ratios (TBRs) for *mIs10* in strains WHR10 (3.86, this study) and PD4793 (6.55, previous study[1]) differed. PD4793 was the original strain obtained from the CGC and is 3x outcrossed. We outcrossed the PD4793 strain to N2 an additional 10 times to obtain WHR10.

We considered four possible explanations for the TBR differences. First, skew TBR is variable, perhaps affected by subtle environmental factors. In this case, the two sets of crosses were conducted in different countries (Taiwan and Switzerland). Second, the tester hermaphrodite strains were different; this study used BRC189 (*unc-119(ed9) III; ttTi5605 II*) while the previous study used CB184(*dpy-13(e184)*). A third possibility is that the *mIs10* insertion allele changed in size. Because there is a correlation between insert size and TBR[1], the presumption would be that WHR10 carries a shorter allele. Finally, there were genetic modifiers that were removed or introduced during outcrossing.

We tested the first two possibilities by reordering the original PD4793 strain from the CGC and then re-assaying skew for both PD4793 and WHR10. Specifically, we crossed heterozygous *mIs10*/+ males to Unc hermaphrodites (BRC189) and scored their progeny. We found that PD4793(new) had a TBR of 6.01 which was not different from the PD4793(old, TBR: 6.55, P = 0.29, χ^2 value = 1.09, 1 df). Similarly, WHR10(new, TBR: 4.33) was not different from the previous WHR10(old, TBR: 3.86, P = 0.19, χ^2 value = 1.65, 1 df). These results indicate that skew TBR is stable and not dependent on the tester strain. For the third case, we estimated insert sizes using qPCR[1] in both strains (4 technical replicates each) and found no difference (P = 0.12, Welch's two-sample t-test = 1.88, df=4.575).

Based on these results, we conclude that one or more skew modifier mutations were removed or introduced during our outcrossing of *mIs10*.

Supplementary Text S2. Insertion size and transmission bias for the interspecies comparisons.

For the interspecies comparisons associated with Supplementary Figure 1, we could not use the same set of reference primers for DNA real-time qPCR due to sequence divergence among the species. Instead, we first identified putative highly conserved elements using the iHCE software package[2] (v4.34; --nompi, all other settings default). For the input dataset, we downloaded the genomes for *C. elegans* (PRJNA13758), *C. briggsae* (PRJNA10731), *C. remanei* (PRJNA53967), and *C. sinica* (PRJNA194557) from Wormbase (all release WS260). From the output, we selected the 9 longest single copy autosomal loci (all >80 bp) for additional sequence inspection across *Caenorhabditis* species using blastn[3,4]. We chose two sequences for preliminary tests based on high nucleotide identity across most species and found that only LG2_6028 produced acceptable qPCR performance (i.e., >95% PCR efficiency and single peak melting curves).

The sequence for LG2_6028 is:
TCAACGGCGCGAGAGATCGGGATAAAGATCGCGTCCGGAACCCATGGTCTGAGTTGAGTGTGTGGTGGTGG
AGGACGGAACCAGGCCAT

We conducted genomic DNA real-time qPCR assays on *bla*[5] (targets the ampicillin resistance marker on plasmids) and LG2_6028 for six strains from four species (*C. elegans, C. briggsae, C. tropicalis*, and *C. brenneri*). We could not test *C. portoensis* because qPCR tests of the reference locus were not reliable. We did not test *C. remanei* because of a repeated bacterial contamination issue.

We calculated copy number using the Pfaffl method that adjusts for PCR efficiency[6] and simplified for single ‘ΔCt’. Then, we estimated transgene sizes by multiplying the ‘unit’ insert size by the approximate copy number. A ‘unit’ was defined as the average of the injected plasmid sizes, except for *mfIs42*, which was the sum of the plasmid and the associated PCR product sizes. Transgene sizes were then normalized to genome size. Finally, we tested for a correlation between the TBRs and normalized transgene sizes using the Spearman’s rank correlation test.

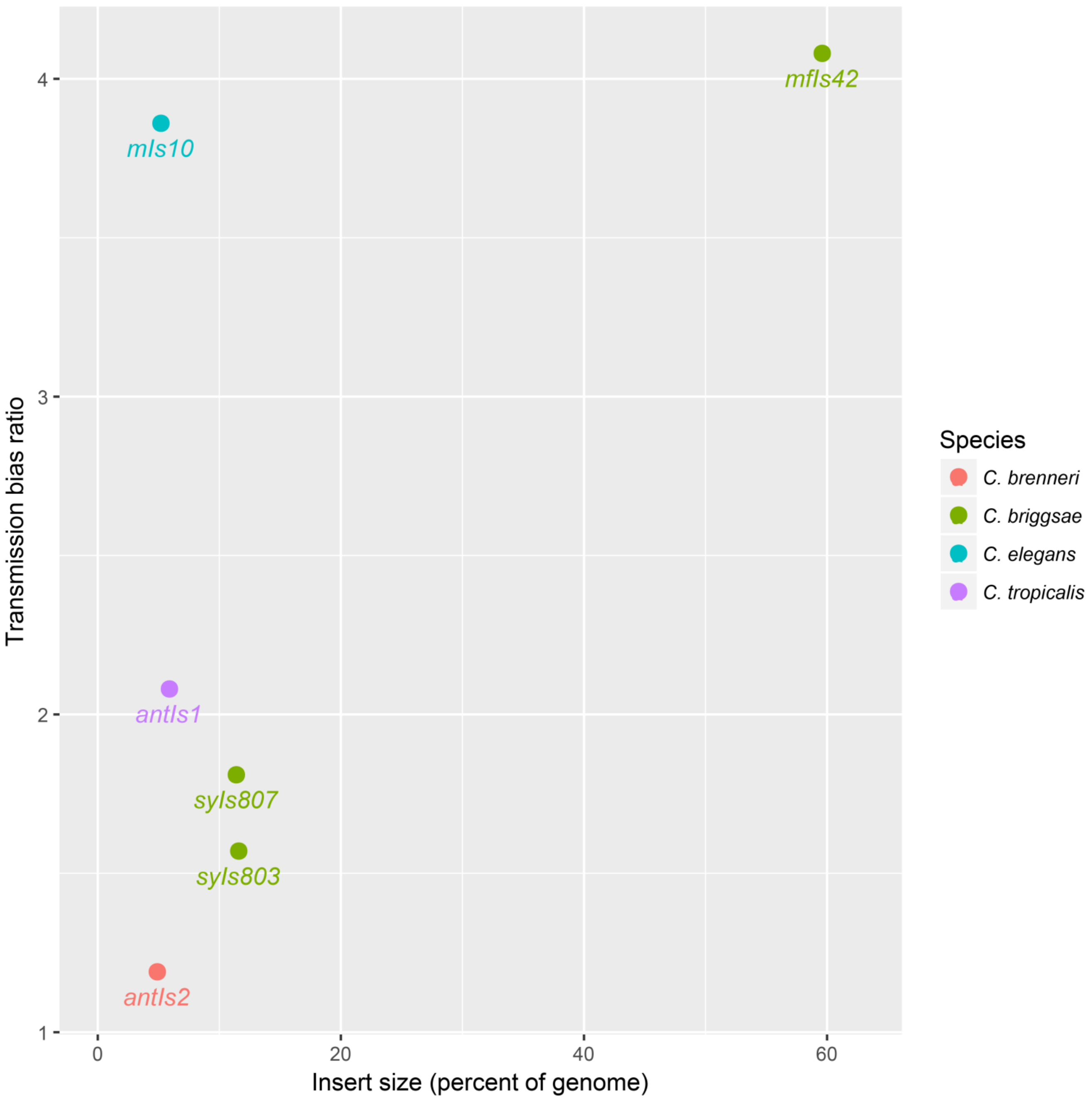

Supplementary Figure S1. Scatter plot of transmission bias ratio and relative insert size. There is no correlation between TBR and insert size across the six samples (P = 0.41, Spearman's rank correlation test) possibly indicating that species-specific factors modify TBR. The three *C. briggsae* data points (green) may be consistent with a within species positive correlation. Transgene names are indicated below in italics.

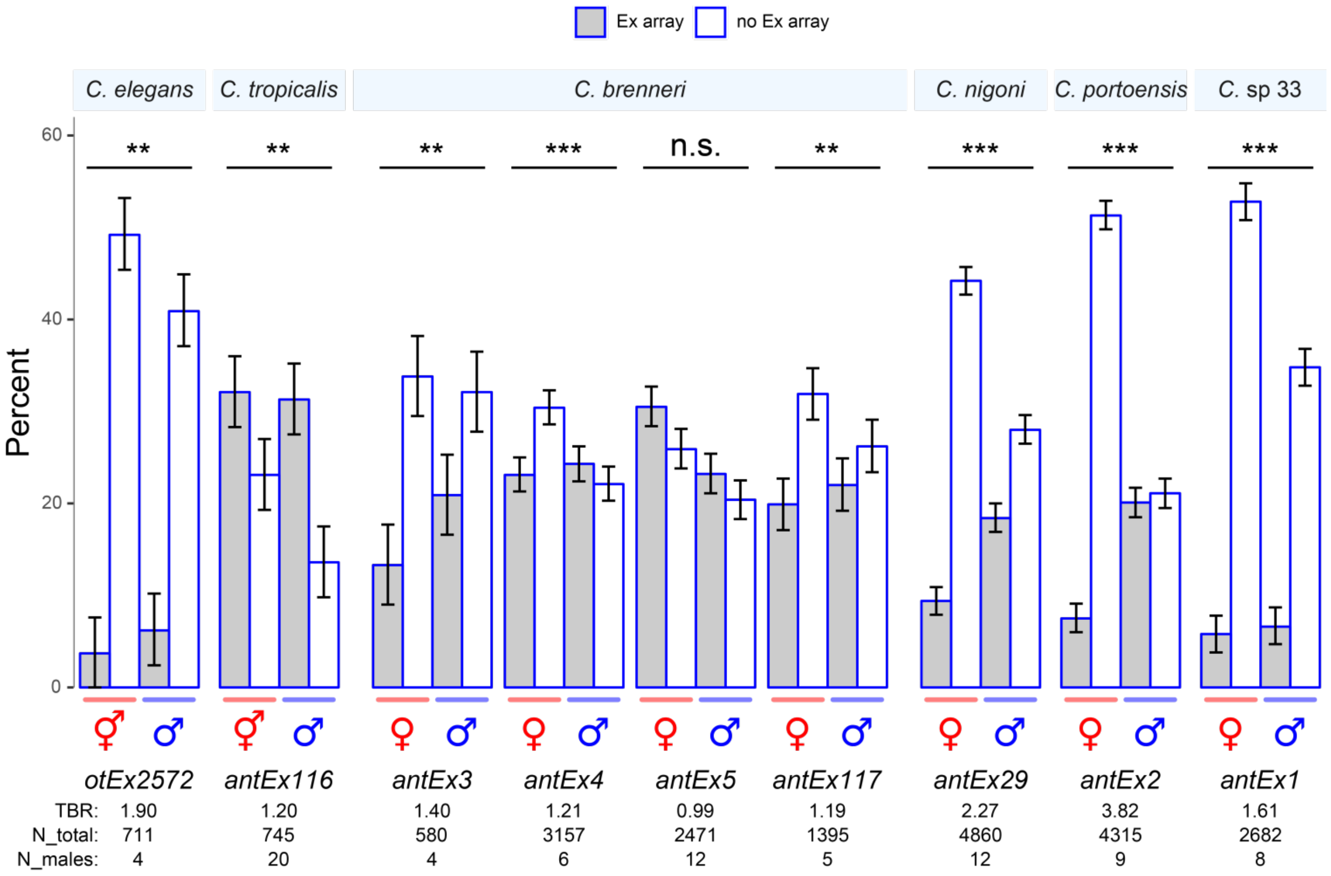

Supplementary Figure S2. Full transmission patterns of extrachromosomal transgenes. Percentages of individuals carrying (gray bars) or not carrying (white bars) extrachromosomal arrays. Species names are indicated above the plots; transgene names are indicated below in italics. Error bars are 95% confidence intervals. *P*-values, χ^2 tests assuming random segregation of the extrachromosomal transgenes by sex; ***, $P < 0.001$; **, $P < 0.01$; n.s., not significant ($P > 0.05$). TBR, transmission bias ratio; N_total, total number of individuals scored; N_males, number of males tested. See Figure 2 for plots of only individuals inheriting the extrachromosomal array. *P*-values are identical between these two figures because the χ^2 tests were conducted on this dataset.

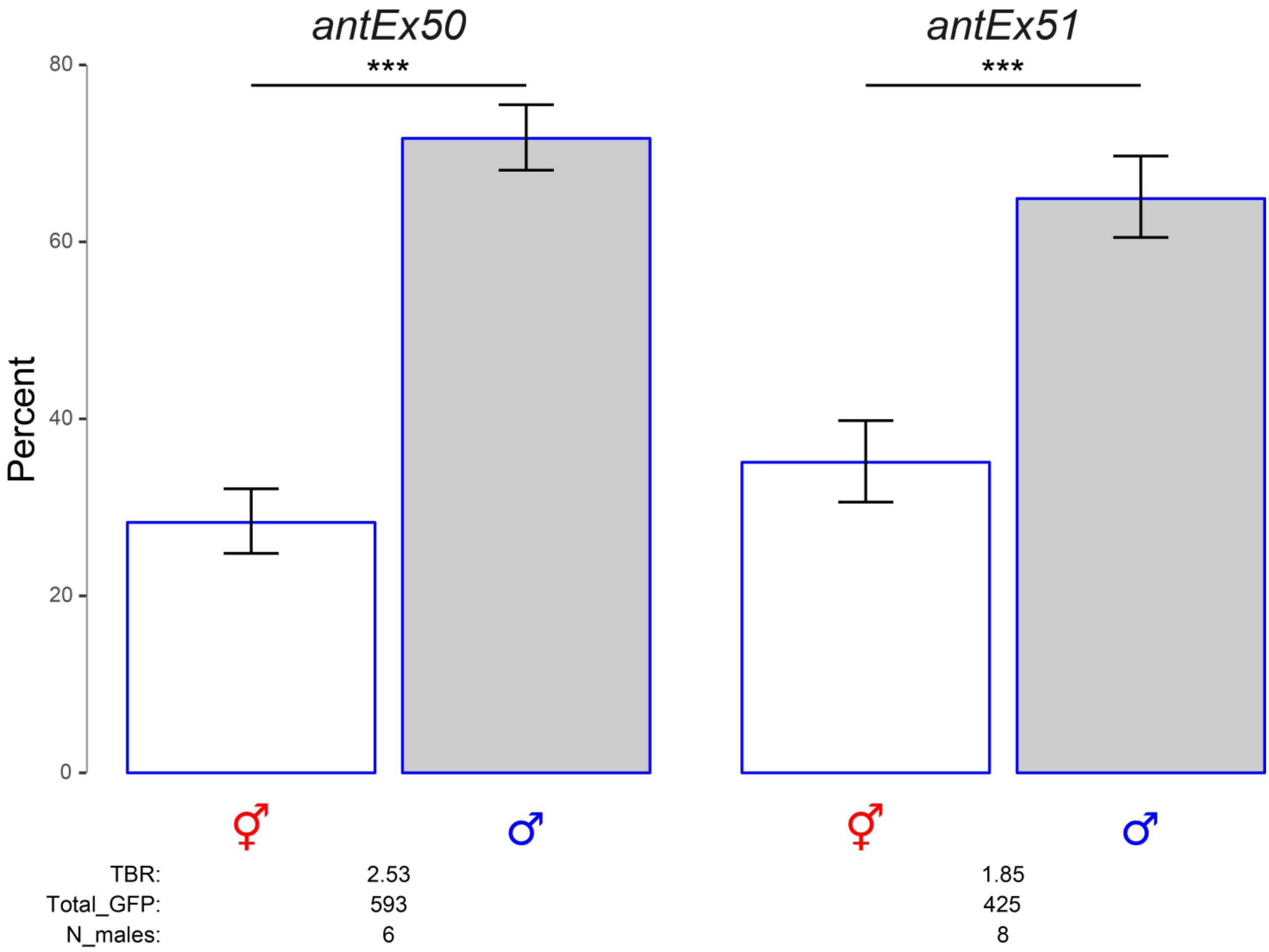

Supplementary Figure S3. Transmission patterns of the *antEx50* and *antEx51* extrachromosomal transgenes.

Percentages of hermaphrodites (white) and males (gray) inheriting the extrachromosomal array. Unc progeny were not scored because self-progeny of the tester *unc-119* strain could not be excluded. Transgene names are indicated above barplots in italics. Error bars are 95% confidence intervals. *P*-values, binomial tests assuming equal segregation of the extrachromosomal arrays by sex; ***, $P < 0.001$. TBR, transmission bias ratio; Total_GFP, total number of GFP individuals scored; N_males, number of males tested.

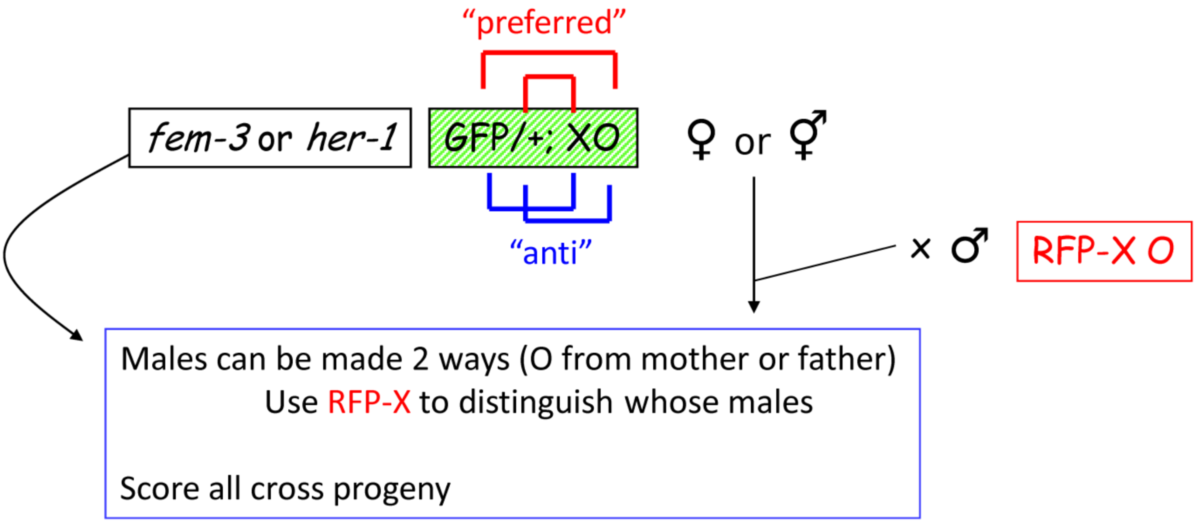

oocyte

sperm	anti	preferred	preferred	anti
	GFP ; X	GFP ; O	non-gfp ; X	non-gfp ; O
X (RFP)	herm	male	herm	male
O	male	dead	male	dead

Supplementary Figure S4. Schematic of *fem-3* and *her-1* oocyte crosses.
fem-3 and *her-1* XO individuals are phenotypically females and hermaphrodites, respectively. Crosses with males carrying an X-linked RFP will produce six viable and two dead progeny classes. Three viable classes correspond to the "preferred" gamete combinations (red box) and the other three classes correspond to the "anti" gamete combinations (blue boxes).

a

RANDOM			oocyte (no skew)			
			1/4	1/4	1/4	1/4
			GFP ; X	GFP ; O	non-gfp ; X	non-gfp ; O
**sperm** (no skew)	1/4	GFP ; X	1/16	1/16	1/16	1/16
	1/4	GFP ; O	1/16	1/16 dead	1/16	1/16 dead
	1/4	non-gfp ; X	1/16	1/16	1/16	1/16
	1/4	non-gfp ; O	1/16	1/16 dead	1/16	1/16 dead

Expected Ratios	XO:XX ratio	
	no skew	if skew at 4:1
GFP/GFP	2:1	5:1
GFP/non-gfp	2:1	2:1
non-gfp/non-gfp	2:1	5:4

Most discriminatory comparison → Transmission bias ratio (TBR) = Observed ratio - 1

if SKEW at 4x			oocyte (no skew)			
			1/4	1/4	1/4	1/4
			GFP ; X	GFP ; O	non-gfp ; X	non-gfp ; O
**sperm** (skew 4:1)	1/10	GFP ; X	1/40	1/40	1/40	1/40
	4/10	GFP ; O	4/40	4/40 dead	4/40	4/40 dead
	4/10	non-gfp ; X	4/40	4/40	4/40	4/40
	1/10	non-gfp ; O	1/40	1/40 dead	1/40	1/40 dead

b

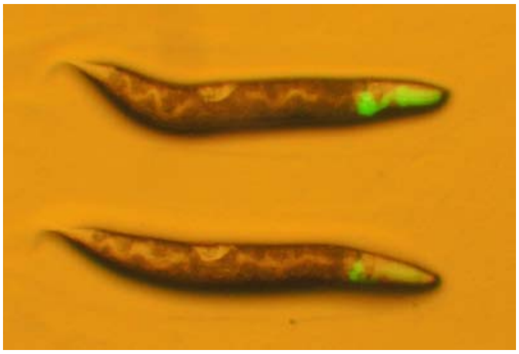

Supplementary Figure S5. *gfp/gfp* homozygotes have the most discriminatory power for the *her-1* sperm test crosses.

a, The top Punnett square illustrates the expected progeny genotype distribution from selfed *her-1 mIs10/++; XO* hermaphrodites with random segregation of chromosomes in sperm. The expected ratio of XO:XX individuals for all three GFP genotype classes (homozygous *gfp/gfp*, heterozygous *gfp/non-gfp*, or homozygous *non-gfp/non-gfp*) is 2:1 (middle). For comparison, the bottom Punnett square illustrates the expected progeny genotype distribution with a skew transmission bias ratio (TBR) of 4:1 in sperm. The expected ratios of XO:XX individuals for the three GFP genotype classes are different (middle), with the homozygous *gfp/gfp* progeny having the biggest XO:XX ratio of 5:1. Thus, the data in Figure 3B focus on *gfp/gfp* individuals. In both models, chromosomes are presumed to segregate randomly in oocytes (see Fig. 3A). The bottom Punnett square can be generalized for any skew TBR (≥1). The relationship between TBR and the XO:XX ratio is: TBR = XO:XX ratio - 1. **b,** Photograph taken through a fluorescence stereomicroscope of *dpy-11 her-1* individuals carrying the *mIs10* transgene either as a homozygote (*gfp/gfp*) or heterozygote (*gfp/+*).

Supplementary Table S1. Information on transgenic strains used in this study

Previously available transgenic strains used in this study

Species	Strain	Transgene	Original Genetic Background	Reference	Notes*
C. elegans	OH4460	*otEx2572 [unc-97::NLS::GFP]*	N2 (wild type)		
	PD4793	*mIs10 [myo-2::gfp, pes-10::gfp, F22B7.9::gfp]V*			
C. briggsae	JU1018	*mfIs42 [Ce-sid-2, Ce-myo-2::DsRed]*	AF16 (wild type)	Nuez and Felix 2012 [ref 7]	
	PS9392	*syIs807 [Ce-daf-4(+), myo-2::GFP]IV*		Inoue et al. 2007 [ref 8]	
	PS9396	*syIs803 [Ce-daf-4(+), myo-2::GFP]II*			
C. remanei	JU1184	*mfIs83 [Cel-sid-2+Cel-myo-2::DsRed]*	PB4641 (wild type)	Nuez and Felix 2012 [ref 7]	8xOC to BRC20108; Previously *mfEx34*

Strains generated in this study

Species	Strain	Transgene	Original Genetic Background	Injected Plasmid (Conc. ng/µl)	Notes*
C. elegans	BRC148	*antEx50 [myo-2::gfp, sur-5::gfp, myo-3::mCherry, rpl-28::puroR]*	BRC150 (*unc-119 (ed3); him-5 (e1490)*)	pDD04neo (5) + pCFJ151-attp-right-sur5::GFP (25) + pCFJ104 (25) + pBCN21-R4R3 (25)	
	BRC153	*antEx51 [myo-2::gfp, sur-5::gfp, myo-3::mCherry, rpl-28::puroR]*			
	WHR10	*mIs10 [myo-2::gfp, pes-10::gfp, F22B7.9::gfp]V*	PD4793		10xOC to N2
C. brenneri	BRC532	*antEx3 [myo-2::gfp, sur-5::gfp]*	CB5161 (wild type)	pDD04neo (20) + pPD158.87 (100)	12xOC to JU1397
	BRC579	*antEx4 [myo-2::gfp, sur-5::gfp]*			10xOC to JU1397
	BRC567	*antEx5 [myo-2::gfp, sur-5::gfp]*			10xOC to JU1397
	BRC536	*antEx117 [myo-2::gfp, sur-5::gfp]*			10xOC to JU1397
	BRC467	*antIs2 [myo-2::gfp, sur-5::gfp]*			7xOC to JU1397
C. nigoni	BRC340	*antEx29 [myo-2::gfp, sur-5::gfp]*	BRC10094 (wild type)	pDD04neo (20) + pPD158.87 (100)	
C. portoensis	BRC313	*antEx2 [myo-2::gfp]*	EG4788 (wild type)	pDD04neo (20)	
	BRC585	*antIs7 [myo-2::gfp]*			6xOC to EG4788
C. tropicalis	BRC493	*antEx116 [myo-2::mCherry]*	JU1373 (wild type)	pCFJ90 + pdestDD04neo	
	BRC555	*antIs1 [myo-2::mCherry]*			3xOC to JU1373
C. sp. 33	BRC311	*antEx1 [myo-2::gfp]*	BRC10016 (wild type)	pDD04neo (20)	

* ##xOC, indicates number of times out crossed to indicated strain

Supplementary Table S2. Primers used for qPCR

Primer name	Sequence	Notes
bla_f1	CTACGATACGGGAGGGCTTA	ref 5
bla_r1	ATAAATCTGGAGCCGGTGAG	
unc-63_qpcr_f1	TGGAATTTGTCACATGGAGGG	ref 1
unc-63_qpcr_r1	TCATCGTGGGCCTACAATTTT	
unc-47_qpcr_f1	TAAGGAGCACCCCTGTCCAG	ref 1
unc-47_qpcr_r1	CAGGTTGGTGGATGGTGGTC	
LG2_6028_F1	GCGCGAGAGATCGGGATA	this study
LG2_6028_R2	CACCACCACACACTCAACTCAGA	

SUPPLEMENTARY REFERENCES

1 Wang, J., Chen, P. J., Wang, G. J. & Keller, L. Chromosome size differences may affect meiosis and genome size. *Science* **329**, 293 (2010).

2 Rubanov, L. I., Seliverstov, A. V., Zverkov, O. A. & Lyubetsky, V. A. A method for identification of highly conserved elements and evolutionary analysis of superphylum Alveolata. *BMC Bioinformatics* **17**, 385 (2016).

3 Altschul, S. F., Gish, W., Miller, W., Myers, E. W. & Lipman, D. J. Basic local alignment search tool. *J Mol Biol* **215**, 403-410 (1990).

4 Camacho, C. *et al.* BLAST+: architecture and applications. *BMC Bioinformatics* **10**, 421 (2009).

5 Lee, C., Kim, J., Shin, S. G. & Hwang, S. Absolute and relative QPCR quantification of plasmid copy number in Escherichia coli. *J Biotechnol* **123**, 273-280 (2006).

6 Pfaffl, M. W. A new mathematical model for relative quantification in real-time RT-PCR. *Nucleic Acids Res* **29**, e45 (2001).

7 Nuez, I. & Felix, M. A. Evolution of susceptibility to ingested double-stranded RNAs in Caenorhabditis nematodes. *PloS one* **7**, e29811 (2012).

8 Inoue, T. *et al.* Genetic analysis of dauer formation in Caenorhabditis briggsae. *Genetics* **177**, 809-818 (2007).
